# The costs of care: An ethnography of care work in residential homes for older people

**DOI:** 10.1111/1467-9566.13546

**Published:** 2022-09-07

**Authors:** Eleanor K. Johnson

**Affiliations:** ^1^ School for Policy Studies University of Bristol Bristol UK

**Keywords:** care, care work, older people, residential care, social care

## Abstract

The cost of social care, the work conditions experienced by care workers and the quality of care provided by residential homes for older people are all linked, yet we know very little about how this relationship works in practice. Drawing upon an ethnography of two differently priced residential care homes for older people in Southern England, I examine the implications of different financial regimes for care‐giving practices. I show how the scheduling and allocation of resources—conveyed, for example, in formal routines and staffing levels—structure the care workers’ time, tasks and activities in each setting. This acts to symbolically demarcate what, or who, is valued. I argue that the availability of resources facilitates and impedes the symbolic culture of care work, shapes care workers’ ability to afford dignity to the individuals in their care and affects how care workers experience, and relate to, their labour. I conclude by discussing how current practices of funding and pricing social care have effects seeping beyond the practical and measurable, and into the realm of the symbolic.

## INTRODUCTION

In the UK, the ageing population, rising ‘care needs’ and growing levels of dependency raise critical questions concerning how, where and by whom (even if) older people’s care needs are being met. It is estimated that 421,100 people aged over 65 years live in care homes in the UK (Age UK, 2018). Homes offer accommodation, meals and twenty‐four‐hour assistance with personal care, including dressing, washing, ‘toileting’ (assisting with elimination needs) and feeding. The term ‘care home’ refers to *residential homes* (the focus of this article), which do not provide nursing care, and *nursing homes*, which have registered nurses on duty at all times.^1^

Concerns have been raised that the supply of social care for older people is not meeting the rising demand (Cottell, 2017). Moreover, as the population ages and people’s needs become more complex, the shortfall in residential care provision is expected to intensify. This growing shortfall emerges alongside reduced public funding for care in austerity Britain (Humphries et al., 2016) and media portrayals focussing on scandals (Lloyd et al., 2014), the working conditions of care workers (e.g., low pay, zero‐hour contracts, high turnover, exhaustion and short staffing) and the financial failure, parsimony or avarice of home providers (Mulley, 2011). The picture painted is of a sector in crisis that is failing older people. Likely to encounter considerable challenges over the coming decades, social care for older people is at a watershed moment. It is vital we continue to subject care and care work to comprehensive academic and empirical inquiry.

I begin this article by outlining key contributions in the literature on care work. From here, I sketch out my study, describing the fieldwork sites and the (ethnographic) research design. In what follows, I examine the implications of different financial regimes for care‐giving practices. I show how the scheduling and allocation of resources—conveyed, for example, in formal routines and staffing levels—structure the care workers’ time, tasks and activities in two care settings. This acts to symbolically demarcate what, or who, is valued. I argue that the availability of resources facilitates and impedes the symbolic culture of care work, shapes care workers’ ability to afford dignity to the individuals in their care and affects how care workers experience, and relate to, their labour. I conclude by discussing how current practices of funding and pricing social care have effects seeping beyond the practical and measurable, and into the realm of the symbolic.

## CARE WORK

Health and social care research is not the province of a single academic discipline. It draws upon a range of frameworks and methodologies from business studies, human resource management and economics to medical and health‐care sciences, public health, social gerontology, policy studies, sociology and anthropology. Yet, common themes and points of congruence emerge. For instance, there is a wealth of research on the quality of care provided to older people (Cameron et al., 2012; Netten et al., 2012). In these studies, ‘quality’ is often measured by either clinical outcome prevalence (e.g., pressure sores, falls, incontinence and mortality) or older people’s own quantitative assessments of their quality of life and wellbeing.

Moreover, there is a growing body of research on paid care work that focuses upon workforce demographics (Yeates, 2009), recruitment, staffing levels and turnover (Hussein et al., 2016) and training and qualifications (Gospel, 2015). There is, also, a considerable literature, and particularly since the early 2000s, that explores how care workers in different settings (such as residential homes, nursing homes and community settings) experience and find meaning in their work (Duffy et al., 2015; Rodriquez, 2014; Stacey, 2005; Twigg, 2000). Such exploration calls for a more qualitative examination of care work, which focuses on the everyday tasks of care‐giving, the perspectives of those undertaking these tasks and their interactions and relationships with the recipients of care. This includes analyses of the emotional elements of care work (James, 1992; Ungerson, 2005), workers’ affective attachment to residents/clients (Berdes & Eckert, 2007), how they deal with challenging behaviours (Roitenberg, 2021), the physical and ‘dirty’ nature of care work (Stacey, 2005; Twigg, 2000) and how, given the often poor working conditions, care workers are motivated by, and find dignity in, their labour (Folbre & Nelson, 2000). In a study on nursing homes, for instance, Lopez (2006) introduces the idea of ‘organised emotional care’ to complement Hochschild’s (1983) well‐used concept of ‘emotional labour’, distinguishable by the presence or absence of organisational feeling rules and affective requirements.

Others have examined the care‐giving relationship and, ultimately, what ‘good care’ looks like. As Folbre and Nelson (2000, p. 129) note, it is widely assumed that it is the possession of caring feelings by a caregiver which both gives them the motivation to carry out care activities and ensures that they provide good care. Studies have shown that, in their assessments of care quality, residents tend to value what they perceive to be particularly moral and motivational dispositions of care workers—such as being ‘genuinely caring’ (Mattiasson & Andersson, 1997; Meagher, 2006, p. 35). Likewise, the relationship‐based elements of care‐giving have often been linked to job satisfaction (Meagher, 2006). It is in this context that the, often tacit, assumption has arisen—both in the academic literature and amongst those operating within the sector—that care workers who genuinely ‘care’ about their clients, and gain intrinsic job satisfaction from doing so, will provide better care than those who are primarily motivated by financial reward (England, 2005). This ‘moral wage’ (Johnson, 2015) is frequently expressed through the ‘family model’ of caring (Dodson & Zincavage, 2007), which stresses the need for care workers to ‘bond’ and establish *authentic* relationships with residents/clients. This can lead to the exploitation of low‐income workers who can become what Folbre (2008, p. 376) has called ‘prisoners of love’.

Whilst I inevitably touch upon similar ideas and findings, I take a different point of departure in this article. Specifically, by focussing on the everyday interactions, routines and rituals of care work in residential homes, I reveal the roles played by political–economic factors, working conditions, material resources and workplace culture in producing particular types of care (and care workers). Scholars have highlighted this political–economic context of social care: its funding, marketisation and commodification (Higgs & Jones, 2009); privatisation and changing patterns of ownership (Drakeford, 2006); competition (Forder & Allan, 2011) and its policy and regulatory context (Haynes, 2007). Yet, this mostly comprises of scoping studies, economic analyses of the social care market and examinations of the social care policy landscape. In short, there is too little empirical exploration that bridges the divide between care quality and outcomes, care work and the wider context of social care.

A small number of scholars have done this, albeit in different contexts of nursing homes. Lowndes et al. (2018) examine how austerity measures resulted in heavy staff workloads that limited the available time to spend with residents. Diamond’s (1992) ethnography is set within a context of wider political, economic and cultural forces in the US which shape and inhibit the quality of care for older residents. In their study on non‐profit nursing homes in Canada, Baines and Daly (2021, p. 385) argue that, within the context of late neoliberalism, care time is ‘political, contested, and multi‐scalar’. They claim that the larger policy context of care work shapes, and ultimately hinders, workers’ capacity to spend time and build relationships with residents. Building on this small but important body of work, I examine how care workers navigate care provision in a context of austerity and a sector that is in crisis—and what impact this wider context has both on the work conditions experienced by care workers and the quality of care provided in residential homes for older people. This moves us beyond human resource‐based analyses of care work and shows how care workers encounter a range of moral, emotional and material stresses that they navigate in diverse ways. My research questions were as follows:

1. What do residents who pay for high‐cost care and those who receive low‐cost care, actually get in the homes they live in? Is there a clear link between the price of care and its quality?
2. What factors contribute to the provision of good and bad quality care? What respective roles are played by management, training, material resources and the normative and symbolic culture of work?
3. What moral, emotional and material stresses are experienced by care workers, and how are these stresses negotiated and managed, both by care workers and their employers?
4. What contribution can sociological theory make to our understanding of the practices and experiences of care workers in residential homes today?

In what follows, I describe the fieldwork sites and research design.

## METHOD

This study was based on an ethnography of two residential care homes for older people. Ethnographies can employ a variety of techniques, yet the method which best allowed me to gain an insider picture of everyday life in the homes was participant observation. Employing an approach which would allow me to take seriously the complex factors which contribute to the provision of good and bad quality care, as well as establishing how these factors were routinely negotiated and managed by care workers and their employers, was fundamental to the aims of my research. An attention to these matters was informed by wider literature, my experience as a care worker and my personal knowledge of the industry, though my inductive and open approach allowed for new observations and ideas to shape the research.

Observations were supplemented with 30 interviews with care workers from other residential homes in the same local authority area. Interviews allowed for an in‐depth examination of the ordinary practices and encounters occurring in residential homes along with a consideration of the moral, emotional and material stresses encountered by care workers. This revealed the respective roles played by political–economic factors; working conditions; material, moral and human resources and workplace culture, among other things, in producing types of care. Interviews with care workers were considered as a useful means to understand the perspectives of those being studied—allowing me to establish the meaning‐making behind what I observed—as well as to further explore the themes which were emerging in my analysis of observational data. My observational data, however, formed the core of my analysis and my subsequent writing, and it is the data that I draw on predominantly in this article.

Over 800 hours of participant observations were carried out over 2 years, where I took on the role of care worker for 12 months, first, at low‐cost Millstead and second, at high‐cost Shorefield (selected because they were at opposite ends of the local authority’s care market). I applied for a position at Millstead and was offered an interview where I was asked where I had worked before, told which tasks I would be expected to carry out and asked when I would be available to start working. Shorefield’s recruitment process was more formal than that at Millstead. I completed an application form collected from the home after I noticed that they had a ‘carer’ vacancy online. Two weeks after returning this form, I was asked to participate in a group interview with three other applicants. I was offered a ‘bank’ care worker (i.e., assigned shifts when an extra care worker was required and I was available to work) position at Shorefield 3 days later. I was also asked to attend a training for this role. I discuss my research design and the ethical issues involved at length elsewhere (Johnson, 2018).

### Millstead and Shorefield

Located in a local authority area in the South of England, Millstead and Shorefield were 1.5 miles apart, but were located in different wards. In the 2010 indices of deprivation, Millstead’s ward was ranked among the top 5% most deprived wards in the UK, whereas Shorefield’s ward was ranked in the bottom 50%. Millstead was a single, private residential home which accommodated 33 residents. It was identified as ‘low‐cost’ due to the low fees that were charged to its 24 local authority and nine private‐paying residents (the average price paid by the local authority for a funded place was £448 per week). Shorefield was a large‐scale corporate home provider. In January 2013, it offered care to 99 residents and the cost of receiving care varied depending on which room a resident occupied and their assessed care needs. The lowest priced fee—including accommodation, meals and activities, but not direct care—was £750 per week. Of the 38 homes in the local authority which formed my initial sample, the cost of care for private payers at Shorefield was the highest, even before taking direct care charges into account. Of the 99 residents at Shorefield, four received funding from the local authority, which paid an average of £540 a week for their care. Shorefield was a purpose‐built home which was marketed as a luxury alternative to more traditional care homes, with advertisements often emphasising its activities, entertainment, cuisine and hotel‐like facilities.

### Data analysis

My iterative approach to data analysis required a constant conversation between theory and data, rather than early formulations of codes that can limit subsequent analysis. I used this analysis to guide areas of future inquiry, whilst being aware of new ideas. Practically, my approach involved (re)reading field notes and interview transcripts, and creating analytical notes. For observations, analytical notes were made alongside handwritten notes in a fieldwork diary. In order to distinguish between data and analysis, a simple process of using different coloured pens was used. This analysis entailed identifying, studying and analysing patterns in the data and noting similarities/differences between observations and interviews with care workers. Ethical approval was granted by the Cardiff University School of Social Sciences Research Ethics Committee.

## FINDINGS

In what follows, I outline the key claims of the article. I describe how the everyday routines of care homes at both ends of the market shine a light on what resources are available to care workers, the impact of economic cost on this and how this shapes the symbolic culture of work required to provide *good quality care*. I focus particularly on three components here: (1) daily routines, (2) the content and philosophies of care and (3) the care worker role.

### Daily routines

The economic cost of care can affect what facilities or services are offered, who these are provided to and the source of payment (together with a home’s revenue, size, demographics and staffing levels). Indeed, appreciating how the provision of care is assessed, costed and paid for allows us to consider the order, content, and pace of care work/care‐giving, and the culture and ethos of a home. Millstead, for instance, conveyed a readiness to house residents with ‘challenging behaviours’, particularly individuals with a recognised history of unsuccessful placements elsewhere. This was often perceived by care workers as a profit‐seeking practice because local authorities are likely to pay more for their care. Notably, for workers, this was not followed by staffing changes, additional training or managerial acknowledgement of their enhanced workload.

At Shorefield, in contrast to Millstead, the cost of residency was directly linked to the amount of care residents were contractually expected to receive; care ‘packages’ were agreed prior to entry with the sales team, formalised in an ‘Individualised Service Plan’ (ISP). In addition, whilst admitting people with high care needs would likely have boosted income, Shorefield’s marketing strategy was not based on securing local authority funding or seeking (profitable) residents with high/complex needs; they were ‘not the Shorefield type’ (Cliff, sales manager). The ‘Shorefield type’ was, primarily, a wealthy individual (or couple) ineligible for publicly funded care provision, both in terms of need and finances, and with no/few care needs.

This all had implications for the amount and type of work undertaken by care workers. Indeed, while the ratio of care workers to residents per shift was alike at Millstead and Shorefield (both in daytime and nighttime shifts), the amount and type of work undertaken by care workers in both homes was significantly different. However, although in part due to variances in residents’ need, it was, also, a result of their different routines (as well as the content/philosophies of care and the care worker role itself).

At Millstead, each day followed a strict routine, organised around residents’ predetermined mealtimes. A basic outline of this routine was displayed on posters placed in communal spaces and staff areas (see Table 1).

**TABLE 1:** Daily routine at Millstead

Breakfast	7:30 AM
Morning tea	10:00 AM
Lunch (early eaters)	11:30 AM
Lunch	12:00 PM
Afternoon tea	2:30 PM
Supper (early eaters)	4:30 PM
Supper	5:00 PM

This conveyed expectations around the timeframe of work. For instance, ‘morning tea’ indicated the target for completing the morning’s personal care activities: ‘toileting’, washing and dressing residents. At Millstead, Mrs G (proprietor) and senior care workers regularly reminded care workers of the fixed nature of deadlines by which they should complete tasks. Care workers occasionally treated the timetable with more flexibility (e.g., when Mrs G was absent), but this could mean bringing forward supper to 4:30 PM and some residents being ‘put to bed’ long before evening (sometimes as early as 4:30 PM). For morning shifts, care workers were responsible for ‘getting up’ either ‘singles’ (residents requiring assistance from one care worker) or ‘doubles’ (requiring assistance from two care workers). Because of short‐staffing, a single care worker would frequently be responsible for assisting all ‘singles’. Likewise, the senior care worker on shift would usually assist with ‘doubles’ and was responsible for dispensing medication, completing paperwork and handover. Usually, the senior and three care workers from the morning shift would stay on for the afternoon shift, working a ‘long day’ (8:00 AM–8:00 PM). In the afternoon, one care worker would be responsible for serving tea/biscuits in the lounge as well as washing up dirty dish, cooking supper, serving in the dining room and doing laundry. The 2 other care workers oversaw the personal care of all 33 residents as well as ‘moving’ them between the lounge, dining room and bedrooms at the times specified in Millstead’s schedule.

Structured around mealtimes, Millstead’s routine classified residents into groups: ‘early eaters’ (residents requiring assistance with eating) and ‘late eaters’ (who did not require assistance). Classifying residents as early/late eaters or singles/doubles was a form of ordering that functioned to divide and routinise work to minimise the effects of short‐staffing (for an analysis of short‐staffing in care homes and its impact on mealtime practices, see Lowndes et al., 2018). Yet, as others show (Hillman, 2014; Jeffrey, 1979; Latimer, 1997), ordering also conveys the value, or lack of, of individuals being cared for. Classification carries a ‘symbolic load’ (Douglas, 1966, p. 4), legitimising a hierarchy of worth and deservingness. At Millstead, the ‘constituting of classes’ (Latimer, 1997, p. 171) conveys not only forms of care but also patients’ identities as dependent and difficult. Some residents even knew their class here; those who ate unassisted frequently denounced the ‘early eaters’ tag and disliked being mistaken for one. Care workers, notably, often expressed a preference for ‘singles’ (who were rarely ‘early eaters’), fuelled perhaps by knowing this meant less physical lifting/manoeuvring, reduced contact with bodily fluids and increased control over the sequencing and pace of their labour.

At (low‐cost) Millstead, then, the limited resources available to care workers—such as time and staff members to share the (work)load with—impacted the working conditions and in turn, the quality of care provided to residents. Strict routines were established, it appeared, to *get the job done*. Yet, this arguably had a damaging impact both on residents (who are denied agency, dignity and dehumanised [e.g., not being referred to by their name, but by meal designation]) and on workers (who were overworked and, as I show below, did not always invest value in their labour).

In contrast, daily routines were flexible at Shorefield. Residents’ needs/preferences dictated the order and nature of care workers’ activities. Each mealtime at Shorefield, for example, was scheduled over the course of two‐to‐three hours to afford residents a degree of choice over when, and where, to eat. Equally, residents could choose when to receive assistance from care workers and how to spend their time. At the beginning of each shift, one care worker would be allocated to each colour‐coded ‘corner’ of Shorefield and would be responsible for residents whose bedrooms were in that area. On morning and afternoon shifts, two care workers would be allocated the role of ‘med tech’, which meant dispensing medication. Every morning, two care workers were allocated ‘breakfast’ (serving in the dining room) and three were ‘floaters’. ‘Floaters’ were tasked with assisting with the care‐giving of residents who required the assistance of two care workers and with answering residents’ pendants (when pressed, pendants sent requests for assistance to care workers’ pagers). In the afternoons, one care worker would be a ‘floater’ and one would be assigned to the dining room.

Flexibility, though, was stressed by both management and care workers themselves. Workers’ time at Shorefield was frequently dictated by the needs/desires of residents who were framed as autonomous consumers and whose satisfaction was a prime responsibility of their designated care worker. Training practices, philosophies and marketing strategies and forms of assigning responsibility also motivated Shorefield’s care workers to advocate on behalf of *their* residents, encouraging each other to provide individualised care. This included an emphasis on workers treating residents ‘as if’ they were family; marketing materials and service principles, for instance, used phrases with familial undertones, focussed on ‘individualised care’ and ‘resident choice’ and borrowed terms from the hospitality industry. My contention, here, is that the flexible schedule and (familial) relationship building, though the latter presents problems (such as the possibility of exploitation; see Johnson, 2015), was made possible by the allocation of resources (such as time and other staff members) at Shorefield. The interplay between the cost of care, the work conditions experienced by care workers and the quality of care provided by residential homes for older people was also made clear in the content, and professed philosophies, of care‐giving provided by care workers.

### The content and philosophies of care

At Millstead, life was frequently fragmented and chaotic. Handovers, where basic information is conveyed about residents between care workers as they enter or leave work, for instance, were informal and muddled. The scheduled number of care workers was rarely reached. The manager (Brian) refused to employ agency workers as they were too costly, so staff were told to ‘spread out’ and ‘make do’ (thereby taking on additional responsibilities/tasks). There was no time or resource dedicated to taking notes (one reason being that several care workers not being able to read/write in English), which meant workers were not aware of residents’ needs, let alone personal preferences, religious affiliations or histories. Priority was placed mostly, if not entirely, on direct care activities. The requirement to save time also had implications for *when* and *how* tasks of personal care‐giving were done. Notably, care workers attempted to save time by moving backward and forward between residents whilst carrying out care‐giving activities. The absence of a detailed division of labour meant that care workers were responsible for all stages and aspects of each resident’s care. The combination of short‐staffing and a high volume of work, however, meant that time did not allow for integrating the many stages of this care‐giving into a single, unbroken process. Rather, the care‐giving process was continually disrupted, stalled and left in temporary abeyance, even abandonment. The following field notes from my first shift at Millstead convey this disorder:

Lidia (care worker) and I are responsible for washing and dressing thirteen residents this morning. Usually (and officially), there would be a third care worker to wash and dress the “singles”, but due to two care workers leaving in the last two weeks, we are short‐staffed. Lidia and I must therefore work together to care for seven “doubles” and six “singles”, as well as making sixteen beds and answering residents’ call bells. It is 8.10am and we must complete this work before 10.30am. Lidia suggests that, in order to complete work at a faster pace, we should hoist more than one resident onto their commodes, work separately to wash each resident, before finding one another to hoist the residents from their commodes into wheelchairs…Lidia and I hoist two residents from their beds onto their commodes and separate to wash and dress each resident…I wash Deidre’s (resident) face, torso, and limbs, but am unable to wash her loins yet, as this will require hoisting…Deidre is ready to be hoisted, washed, and transferred into her wheelchair, but there is no sign of Lidia or the hoist. I apologise to Deidre and leave her room to find Lidia. She is not in Jim’s room, where I left her, but Jim, like Deidre, is half‐washed and half‐dressed and sat on his commode. After checking several residents’ bedrooms, I find Lidia with Judith. Judith is bed‐bound…two care workers must assist with her personal care because she requires turning. Lidia has started washing the parts of Judith’s body which she can reach. As I enter Judith’s bedroom, the call bell starts to ring loudly in the hallway, where a room number is displayed on a screen. Geoff, whose room is upstairs, asks me to help him onto his commode. Another resident presses the call bell as I slowly walk with Geoff towards his commode, but I am unable to answer it. When I have finished with Geoff, I return to help Lidia with Judith, but she has left Judith, half‐clothed, in order to answer the other call bell. When Lidia returns, we return to “the doubles” to complete their personal care.

At Millstead, life was hectic and unpredictable, leaving care workers with little option but to muddle through tasks as quickly, and pragmatically, as possible. Residents would regularly be left halfway through direct (personal) care‐giving, until two care workers were available to hoist or turn them. Meanwhile, call bells rang continuously. Residents pressed call bells when requiring assistance, and the result was a loud, repetitive alarm sounding until the bell was answered. Most often, care workers would be otherwise occupied when the alarm sounded, and the result was that care workers would rush backwards and forwards between residents, commonly to deactivate the alarm. The answering of residents’ call bells was one of the few daily tasks at Millstead not amenable to the daily routine: it was unpredictable and unquantifiable. As such, care workers frequently complained when residents pressed their call bells, made clear to residents that they were felt to be a burden and attempted to dissuade them from requesting assistance again.

When tasks increased, more time‐saving measures were introduced by care workers: Jennifer and Erica (senior care workers) often hoisted residents alone if they were unable to find their ‘double’; Sorin and Mahesh would attempt to implicitly persuade residents to say they did not need to be washed or claim that they had been given a ‘thorough’ wash yesterday (particularly if the resident was forgetful). In such cases, washes became quicker and less thorough, soap was not rinsed off and having privacy when using the commode (a normal expectation at Shorefield) was a privilege only granted when care workers were summoned elsewhere. One less harmful way to save time at Millstead would have been to reduce the time spent on housekeeping tasks like bed‐making. Puzzlingly, this was the one time‐saving measure care workers were reproached for by the management, perhaps because it was the one method with an aesthetic impact clearly visible to visitors and inspectors. In such moments, the *appearance* of care was regarded as just as, if not more, important than the *substance* of care (Killett et al., 2016).

Despite the lack of time for care workers to complete all of their allocated tasks and activities, there were also periods when it was less busy. Afternoon shifts were normally quieter than mornings, yet this available time was not usually spent caring for, or talking, to residents. During afternoons, I was often told by senior care workers and care workers to ‘look busy’ or to ‘just wander around’. Care workers were not permitted to sit in the lounge and, when I talked to residents, I was regularly told that I needed to ‘find some work’. Care workers, thus, would simply pace the corridor, popping in and out of bedrooms which they knew were empty as though looking for someone or something. Another strategy was to keep out of sight completely. Care workers would often fill time by taking long trips to the toilet located upstairs or by claiming they were caring for a bed‐bound resident, when, in reality, they were eating a snack or using their phone in an empty bedroom or, even, in the corner of a bedroom where a resident was confined to bed. One constant was that ‘finding work’ never entailed spending more time with residents.

Life at Shorefield, in contrast, was less hurried, not least due to a flexible schedule adhering to residents’ pace and preferences, lower resident needs and an overestimation of required care‐giving. Personal care took time (sometimes over an hour with each resident) and this was rarely fragmented, sped up or split between residents. Consider the act of care‐giving for Beatrice (resident) which lasted over an hour and involved Helena (care worker) and I carrying out both physical and emotional labour:

Helena goes to collect the hoist from the storage cupboard and says she will meet me in Beatrice’s room. I head to Beatrice’s room, wish her a good morning, and ask her if she would like a bed‐bath or shower. Beatrice decides upon a bed‐bath. I prepare two bowls of warm water, wipes, gloves, and an incontinence pad. Whilst I am in Beatrice’s *en suite*, Helena knocks and enters Beatrice’s bedroom and asks her what she would like to wear. We stand on either side of Beatrice’s bed and undress her. Helena wipes her down with a soapy flannel, followed by a non‐soapy flannel, and then I use towels to dry Beatrice’s arms, legs, and chest. Beatrice tells us stories about her GP career whilst, turning her from side to side, we wash and dry her buttocks. We apply creams and perfume to Beatrice’s body before dressing her in her chosen clothes, explaining with each step what is happening. We hoist Beatrice into her armchair. Helena boils the kettle and assists Beatrice with inserting her dentures and applying make‐up whilst I clean up and make Beatrice’s bed. “Homes Under the Hammer” is on television and Beatrice tells us how sad she is that she has had to sell her house. We sit and comfort her for around five minutes, asking if there is anything we can do. Beatrice thanks us and hugs each of us, joking we should live with her. We tell Beatrice we will come back to make her another cup of tea soon.

Time with residents at Shorefield was never rushed or monitored. Caring well involved talking to residents along with carrying out physical tasks and was adapted to everyone’s needs and preferences. When there were few physical direct care tasks to perform (particularly in the afternoons when residents participated in activities), care workers would usually sit and talk to residents or take hot drinks and snacks to residents’ bedrooms. In contrast to Millstead, Shorefield’s care workers were explicitly, and actively, urged by the management to use any available time at work ‘getting to know’ residents. Moreover, care workers spent lots of time writing notes, completing forms and reading information about residents. Handovers involved exchanging information, documentation, using medical language, and abiding by procedural regulations. I argue that such practices allowed for the cultivation of a collective professional identity that motivated workers to have a shared sense of responsibility for individuals in their care (Meagher, 2006). This not only improved the quality of care for residents, but also allowed workers to find value in their labour (e.g. providing opportunities to develop relationships with residents) – in ways that did not seem possible in the frenzied, capricious, and frequently overwhelming environment at Millstead. Finally, the relationship between the economic costs of care, work conditions and the quality of care was evident in what labour care workers at Millstead and Shorefield were expected to undertake.

### Carers, cooks, or cleaners? The care worker role

At Millstead, there was a simple division of labour that meant care workers undertook several tasks during each shift:

It’s 3.40pm. I have been asked to walk to the shops to buy packs of potato waffles for the residents’ supper, which Henrietta (care worker) and I must prepare before 4.00pm. Care workers are responsible for cooking supper every day, since the cook leaves at 1pm, but sometimes there are not enough ingredients in the kitchen. Brian (manager) seems to be adding tasks outside of direct care activities to our job role more frequently…This week, I have worked as a kitchen porter, cook, cleaner, bed‐maker, launderer, and I have been asked to chop vegetables for Mrs G’s [proprietor] private dinner party.

The boundaries between care work and other kinds of work were blurred at Millstead. Care workers undertook duties usually undertaken by other low‐paid workers (e.g., cleaners and kitchen assistants), including washing, drying, sorting, folding and returning clothes to residents’ bedrooms; tidying storage cupboards; unpacking food and linen deliveries; restocking gloves and paper towels; peeling vegetables; making breakfasts and suppers; serving meals; cleaning the kitchen; cleaning the dining room after each meal; washing up; making and changing beds; cleaning toilets and commodes and emptying bins. In addition, just as care workers were expected to double as cooks and cleaners, so too Millstead‐employed cleaners would frequently be asked to ‘fill in’ as care workers (despite no formal training in care‐giving). Whilst the tasks of ‘care work’ were blurred, so too was care workers’ definition of what/who a care worker was and what/who the subject of their labour was.

During fieldwork, I was able to observe several instances where, instead of explicitly defending or advocating for the boundaries of the care worker role, care workers typically behaved in ways that were at the expense of older persons, such as taking a (not‐sanctioned) break. A superficial observation may see Millstead’s care workers’ acts of resistance as ineffective and petty or, worse, as selfish. It was not long after I started working at Millstead, though, that I realised the preciousness of having moments to yourself and, even, having time to eat. There was one occasion when, having worked for over 9 hours without eating, I ate a resident’s uneaten sandwich in the lift (elevator). Here, moments of resistance (to taking on more work traditionally beyond the care‐giving role) were often degrading.

In contrast, the role of the care worker at Shorefield was clearly demarcated. Workers had a strong sense of worker identity and made a more concerted, collective effort to defend the specificity of their jobs. Ideas about what was, and was not, part of the care worker role were facilitated by Shorefield’s complex division of labour. Most cleaning tasks were undertaken by housekeepers, but care workers were, at the start of fieldwork, responsible for conducting morning ‘room checks’ on bedrooms not scheduled for housekeeping. This involved emptying sanitary waste bins, making beds and cleaning sinks and toilets. Six months later, room checks became the housekeepers’ responsibility. Another 3 months later, after housekeepers constantly informed Shorefield’s management that they were overworked, room checks were passed back to care workers. The following field note documents were the care workers’ resistance to this:

Room checks usually take less than five minutes per bedroom, but care workers are assigned up to twenty‐three bedrooms on a shift and, sometimes, all of these rooms must be checked by an individual care worker. I have been told by Jade (care worker) to “just not do a very good job of it” when making beds. The care workers are aware that several housekeepers are meticulous when checking the residents’ bedrooms and believe that doing a poor job…will result in room checks being made the housekeepers’ responsibility again. This appears to be working: several messages from housekeepers regarding “how to do hospital corners”, “who likes their bed sheets tucked in”, and “when to change sheets” have been passed on in handover. Each time, the care workers respond with: “we just don’t have time to do it properly”.

Care workers’ attempts to take control over their work were successful: several weeks later, room checks were again made the responsibility of housekeepers. Here, care workers enacted small acts of separation to distinguish what they perceived as ‘proper’, legitimate and valued care work and what was inappropriate to their role. Along with close relationships with colleagues, it allowed care workers to carve out a positive (expected) professional identity. Whilst, for Millstead’s care workers, resistant acts were more demeaning than empowering; for Shorefield’s workers, acts of resistance (like not doing a ‘very good job’ of making beds) allowed for greater control of the boundaries of their work. Indeed, at Millstead, issues such as short‐staffing meant that care workers often performed tasks that were not part of the usual care worker role and that this, inevitably, had a negative impact on the quality of care offered to older residents—symptoms, I contend, of an anomic working environment. Moreover, such issues did not allow for a collective, cohesive professional identity among workers and the establishment of a clear moral economy. Without the necessary resources, care workers rarely found joy or value in their labour, nor did residents always receive care that afforded them dignity, belonging and respect.

## DISCUSSION

The major impulse behind this study was a concern that care in later life is subject to wide variations in quality and that older people who are reliant on public funding or subsidising of their care are at a much greater risk of receiving poor quality care. By examining the everyday work practices of care workers at low‐cost Millstead and high‐cost Shorefield, my intention was to consider what residents were receiving in terms of the quantity and quality of care. Moreover, I wanted to establish what such care—whether good, bad or unexceptional—rested upon. What roles were played by management, training, material resources and the normative and symbolic culture of work, for example, in (good) care provision? Since previous research has indicated that the quality of care is linked to the quality of work conditions in residential homes, my pursuit of answers to these questions required that I examine the moral, emotional and material stresses that are experienced and managed by care workers.

In this article, I highlighted and unpacked the interplay between the cost of care, conditions of work and the quality of care provided for older people. By focussing particularly upon daily schedules, the institutional philosophies of Millstead and Shorefield and how the care worker role itself is configured and enacted, I show how care work, and notions of good quality care, are affected by what normative resources—both material (e.g., staffing levels and equipment) and symbolic (e.g., carving out time to perform duties and to build relationships/establish a strong sense of community)—are available.

To be clear, I depart from problematic conceptions of care as that leaving a recipient feeling ‘recognised and valued as an individual, emotionally supported, emphatically connected, or in shorthand, loved’ (Folbre & Nelson, 2000, p. 129). In a privatised care sector, the promotion of ‘family‐like’ care can work to ‘institutionalise an expectation of self‐sacrifice’, which creates a ‘workplace culture ripe for the exploitation of…care workers’ (Dodson & Zincavage, 2007, p. 922)^2^. Instead, I propose using more normative resources to evaluate the quality of care and how this might be delivered without ignoring or sacrificing the rights and humanity of care workers.

Equally, I am not saying here that more economic resources result in good (/*better*) care. This article, and particularly the case of Shorefield, has shown that good quality care is rooted in symbolic systems—and that cultivating and maintaining a culture of good care, which allows and encourages care workers to convey moral regard and respect for individual residents and each other, requires the presence of particular resources. This includes, as I argue elsewhere (Johnson, 2018), attending to matters, including language, materiality and community building. In this article, I particularly highlight how time underpins good quality care. Those who have expressed concerns about the quality of residential care have recognised the impact that time pressures have upon care workers’ ability to do a good job (Kemper et al., 2008; Lee‐Treweek, 1997). The amount of time that care workers are able to spend undertaking care activities is directly linked to staffing levels (Eaton, 2000). Here, I also reveal two further casualties of the lack of time caused by short staffing: care workers’ sensitivity to interpersonal communication and their capacity for symbolic work. Forced to complete many (and largely, if not exclusively direct/physical) tasks in a tight schedule, care workers in short‐staffed homes have little time for the luxury of acting with ‘demeanour’ or communicating ‘deference’ to those in their care (Goffman, 1956).

When care workers are pushed for time, they are left with little option but to prioritise certain aspects of their work and eliminate others; frequently here, the ‘invisible’, emotional and immeasurable aspects of care, largely, became the first to be relinquished (Diamond, 1992). Moreover, when time is in short supply, the ‘hands on’ tasks of care—toileting, washing and dressing—are undertaken in the quickest time possible. This speed‐up results in a routinised, factory‐line form of care‐giving, where residents are treated not as respected individuals, but as dehumanised products on an assembly line. My observations at Millstead revealed the type of care work observed by Lee‐Treweek (1997: 57)—which ‘consisted…of acts performed on objects in the swiftest way possible’—is still taking place in residential homes in England. What I add to this knowledge is an understanding of how, in a commodified care sector, the relationship between individualised care and time plays out in different ways at different ends of the care market. Interestingly, as stated above, the care‐worker‐to‐resident ratio in Millstead and Shorefield during the daytime was fairly similar; during the night, Millstead’s ratio of care workers to residents (1:17) was actually higher than at Shorefield (1:22). My study, thus, makes clear that future research and Care Quality Commission (CQC) inspectors must examine and regulate for other factors that have implications for how much time is available for care workers to undertake care activities: the quantity and quality of residents’ care needs; the complexity of a care home’s division of labour (e.g., if care workers are also required to cook and/or clean) and how time is allocated to individual residents. As Baines and Daly (2021, p. 385) claim, time ‘commodifies, disciplines, and delimits workers’ experience of care, and fractures human relations and solidarities’.

This is a story, then, of the impact of financial regimes on the quality and equity of care for older people and of how care workers subsequently experience (and come to [de]value) their labour. The availability of normative resources necessary for delivering good quality care, as the cases of Shorefield and Millstead shows us, is increasingly entwined with the costing and funding of care. The current funding and regulation of residential care is not working to ensure good quality care for *all* older individuals. Current practices of funding and pricing social care have effects that seep beyond the practical and measurable, and into the realm of the symbolic. It is only by taking the normative and symbolic culture of work in residential homes seriously that we can fully recognise and work towards establishing a care sector which is equitable, both for older people and for those who take care of them.

## Data Availability

Research data are not shared.

## ENDNOTES

^1^
In certain places, such as North America, the terms ‘residential care homes’ and ‘nursing homes’ are used simultaneously when referring to the same facility. In this article, I report on my study in *residential* care homes (where nursing care is not offered). Both homes that were selected as my field sites, Millstead (a low‐cost home) and Shorefield (a high‐cost home), were part of the private residential care market. The focus on private, rather than public, residential homes was principally because all residential homes in the local authority area—meaning an organisation that is officially responsible for all the public services and facilities in a particular location—were operated by either private or non‐profit providers. It is worth noting that whilst Shorefield has a higher cost than Millstead, some residents at both care homes received funding from the local authority (although, in the case of Shorefield, relatives had to ‘top up’ the cost of their care). For more details on the financing of both homes, see Johnson (2018).

^2^
Interestingly, Shorefield’s care workers were explicitly and actively urged by management to use any available time at work ‘getting to know’ residents. Shorefield’s encouragement of teamwork and family‐like relationships had an ethical dimension, and it could be seen to benefit residents, but a consequence was that the boundary between work and home was blurred. As care at Shorefield is predominantly undertaken by women, and supposedly out of love or moral/social obligation, blurring the home/work boundary arguably made it harder for female care workers to recognise and mark out the boundaries and economic value of their labour (Johnson, 2015). Thus, we should avoid painting a too rosy picture of this urge for intimacy and must approach such recommendations with caution. For a further critique of the familial model of care, see Dodson and Zincavage (2007), Johnson (2015) and Meagher (2006).
